# Vancomycin-associated acute kidney injury: A cross-sectional study from a single center in China

**DOI:** 10.1371/journal.pone.0175688

**Published:** 2017-04-20

**Authors:** Kunming Pan, Lingyun Ma, Qian Xiang, Xueying Li, Haixia Li, Ying Zhou, Li Yang, Yimin Cui

**Affiliations:** 1 Department of Pharmacy, Peking University First Hospital & College of Pharmacy, Peking University Health Science Center, Beijing, China; 2 Department of Pharmacy, Peking University First Hospital, Beijing, China; 3 Department of Biostatistics, Peking University First Hospital, Beijing, China; 4 Department of Clinical Laboratory, Peking University First Hospital, Xicheng District, Beijing, China; 5 Department of Nephrology, Peking University First Hospital, Xicheng District, Beijing, China; Bambino Gesù Children's Hospital, ITALY

## Abstract

**Objective:**

The objective of this study was to investigate the current situation of vancomycin (VAN)-associated acute kidney injury (VA-AKI) in China and identify the risk factors for VA-AKI, as well as to comprehensively examine the risk related to concurrent drug use. Further, we assessed the outcomes of patients who developed VA-AKI and the risk factors for these outcomes. Finally, we aimed to provide suggestions for improving the prevention and treatment of VA-AKI in China.

**Methods:**

We conducted a retrospective observational study of inpatients who had been treated with VAN between January 2013 and December 2013 at Peking University First Hospital. AKI was defined as an increase in SCr of ≥0.3 mg/dl (≥26.5 μmol/l) within 48 hours or an increase to ≥1.5 times the baseline certainly or presumably within the past 7 days. VA-AKI was defined as the development of AKI during VAN therapy or within 7 days following the termination of VAN therapy. In addition, we compared patients with NO-AKI, who did not develop AKI during their hospitalization, with those with VA-AKI.

**Results:**

Of the 934 patients treated with VAN during their hospital stay, 740 were included in this study. Among those excluded, 38.1% (74/194) were excluded because of a lack of data on serum creatinine (SCr). Among the included patients, 120 had confirmed VA-AKI, with an incidence of 16.2% (120/740). Multiple logistic regression analysis revealed that an elevated baseline estimated glomerular filtration rate (eGFR) (odds ratio [OR] = 1.009; p = 0.017) and concomitant vasopressor therapy (OR = 2.942; p = 0.009), nitrate use (OR = 2.869; p = 0.007), imipenem-cilastatin treatment (OR = 4.708; p = 0.000), and contrast medium administration (OR = 6.609 p = 0.005) were independent risk factors for VA-AKI; in addition, the receipt of orthopedic/trauma/burn surgery (OR = 0.3575; p = 0.011) and concomitant compound glycyrrhizin use (OR = 0.290; p = 0.017) were independent protective factors for VA-AKI. Multiple logistic regression analysis also demonstrated that among the patients who developed VA-AKI, coronary heart disease (CHD) (OR = 12.6; p = 0.006) and concomitant vasopressor therapy (OR = 15.4; p = 0.001) were independent risk factors for death. We also evaluated the factors influencing improvement of renal function. Multiple logistic regression analysis demonstrated that CHD (OR = 8.858, p = 0.019) and concomitant contrast medium administration (OR = 9.779, p = 0.005) were independent risk factors and that simultaneous β-blocker treatment (OR = 0.124, p = 0.001) was an independent protective factor for improvement of renal function.

**Conclusion:**

Patients treated with VAN received insufficient monitoring of SCr and inadequate therapeutic drug monitoring. We recommend that hospitals increase their investment in clinical pharmacists. An elevated baseline eGFR and concomitant vasopressor therapy, nitrate use, imipenem-cilastatin treatment, and contrast medium administration were independent risk factors for VA-AKI; in addition, orthopedic/trauma/burn surgery and concomitant compound glycyrrhizin use were independent protective factors for VA-AKI.

## Background

Vancomycin (VAN), a glycopeptide antibiotic, was discovered in the 1950s. The prescription of VAN has increased in parallel with the rising prevalence of invasive methicillin-resistant *Staphylococcus aureus* (MRSA) infection [1–3]. Given the increased resistance of deep-seated infections to VAN, clinical guidelines suggest targeting trough VAN levels of 15–20 mg/l for such cases to ensure clinical efficacy and avoid adverse drug reactions (ADRs) as much as possible. However, acute kidney injury (AKI) is still the main serious ADR experienced by patients receiving VAN treatment, and it can threaten patients’ health and even their survival. Some studies have reported AKI incidences of up to 40% [4,5]. Thus, it is clinically important to confirm the risk factors for VA-AKI to help guide clinicians toward the more rational administration of VAN, with increased renal safety and the avoidance of AKI development. Currently, many factors are known to affect AKI development. Patients who are older, receiving therapy for a long duration and/or receiving therapy concomitantly with nephrotoxic agent treatment, have a high trough level of VAN, are critically ill and have compromised renal function prior to VAN treatment are at particularly high risk of VAN-induced nephrotoxicity [5]. However, relevant findings have not been consistent between among studies [6], and there may be ethnic differences in these risk factors [6]. To date, studies of the Chinese population are very limited, and little is known about the risk factors for VA-AKI among Chinese individuals. A recent national survey on AKI in China has revealed a serious condition issue with drug safety in the AKI population, as up to 70% of AKI patients had been exposed to potentially nephrotoxic drugs before or during their kidney injury. This finding raises a major issue, indicating that the healthcare system should increase the renal safety of drug treatments in China [7]. Drug combinations have complex effects on renal toxicity; however, relevant studies are very limited. Several studies have shown that concomitant administration of amphotericin B, tobramycin or tacrolimus, vasopressor agents or intravenous contrast medium may increase the risk of VA-AKI [5], although few drug combinations were examined in these studies.

The objective of this study was to investigate the current situation of VA-AKI in China and to identify the risk factors for VA-AKI in the Chinese population, as well as to comprehensively analyze the potential impacts of drug combinations, including Chinese patent medicines. Further, we examined the outcomes of patients who developed VA-AKI and the risk factors associated with these outcomes. Finally, we aimed to provide some suggestions for improving the prevention and treatment of VA-AKI in the Chinese population.

## Methods

### Study design and population

This was a single-center retrospective study performed at Peking University First Hospital. We recruited all patients treated with VAN at our hospital from January 2013 to December 2013. Written informed consent given from the patients whose records were used was not given. Because our study was a retrospective research and data was based on discharged patients medical records. The consent procedure approved by the Ethics Committee that was approved this study. This study was approved by the Peking University First Hospital Clinical Research Ethics Committee on December 15^th^, 2015. The Approval number was 2015 (998] (see S1 File). Patient data was anonymized prior to analysis. Another pharmacist who was not participated in this study was responsible for anonymizing patient data.

### Survey design

**Fig 1:**
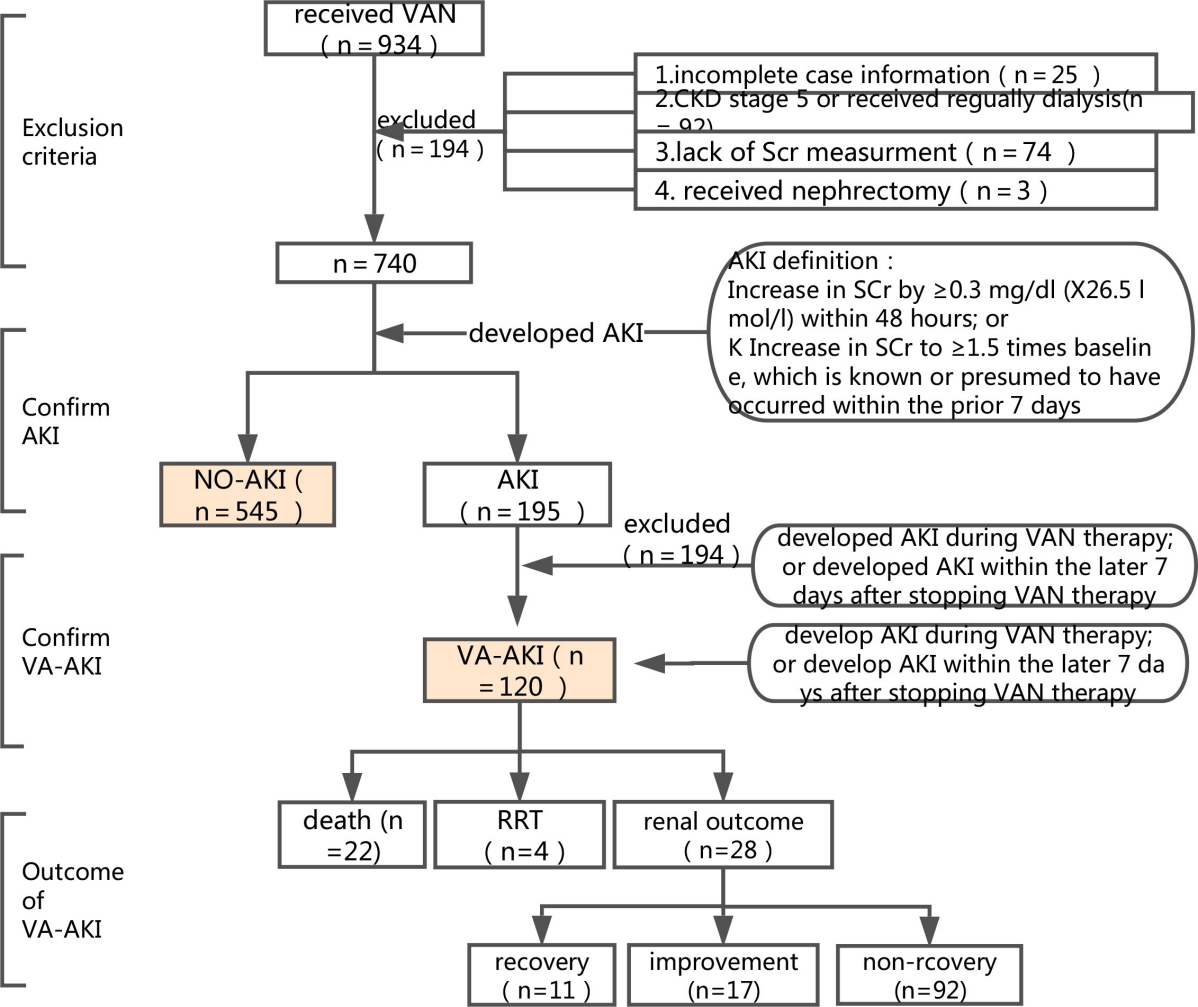
Survey design.

The survey of AKI was designed to include four steps (Fig 1). First, we screened the patients treated with VAN at our hospital; all patients ≥18 years old were included. Patients were excluded if 1) their medical records were incomplete; 2) they had been diagnosed with stage 5 chronic kidney disease (CKD) or were regularly receiving dialysis; 3) their serum creatinine (SCr) were not being adequately monitored to detect the development of AKI; or 4) they had undergone nephrectomy.

Second, we recorded the SCr of the included patients and separated the patients into two groups according to the definition of AKI: the NO-AKI group and the AKI group. We used the 2012 Kidney Disease: Improving Global Outcomes (KDIGO) definition of AKI as the major screening criterion [8]: increase in SCr by ≥0.3 mg/dl (≥26.5 μmol/l) within 48 hours or increase in SCr to ≥1.5 times baseline which is known or presumed to have occurred within the prior 7 days.

Third, we further excluded patients who developed AKI before receiving VAN treatment or more than 7 days after stopping treatment. The definition of VA-AKI was defined as the development of AKI during VAN treatment or within 7 days following the termination of VAN treatment.

Fourth, for the patients who developed AKI, we further analyzed the development, severity and outcome of AKI. AKI severity was described by the highest stage of AKI (1, 2, or 3) and receipt of renal replacement therapy (RRT), according to the KDIGO criterion. AKI outcome was examined using three variables: all-cause in-hospital death, receipt of RRT and renal outcome at discharge. Renal outcome was categorized into three levels: recovery, improvement and non-recovery. Recovery was defined as restoration of the SCr during hospitalization to the baseline; improvement was defined as a decrease of at least 25% in SCr during hospitalization from beginning of AKI onset; and non-recovery was defined as lack of improvement in SCr at discharge. We combined the recovery and improvement groups into one group for examination of the risk factors related to the renal outcome of VA-AKI.

### Data collection

We collected the following variables: demographic characteristics (gender, age, and weight), admission department (medical, surgical, and ICU), concomitant diseases (hypertension, diabetes, coronary heart disease [CHD], CKD, and chronic lung disease [CLD]), laboratory data (baseline SCr), reason for VAN treatment (prophylactic, local infection, and bacteremia), hospitalization factors (length of stay [LOS], ICU admittance, cancer, and surgery), source of VAN, length of therapy (LOT), average daily dosage, therapeutic drug monitoring (TDM), and the use of concomitant drugs (vasopressors, nitrates, β-receptor blockers, phentolamine, amlodipine, sodium nitroprusside, angiotensin II receptor blockers (ARBs), angiotensin II-converting enzyme inhibitors (ACEIs), aminoglycosides, amphotericin B, azole antifungals, micafungin, metronidazole, nystatin, acyclovir, imipenem-cystatins, β-lactam antibiotics, macrolides, quinolones, sulfonamides, tigecycline, diuretics, 20% mannitol, low-molecular-weight dextran, nonsteroidal anti-inflammatory drugs (NSAIDs), immunosuppressants, glucocorticoids, chemotherapy drugs, compound glycyrrhizin, compound fresh bamboo juice, liquorice tablets, Ganmao Qingre granules, Yunnan Baiyao capsules, ursodeoxycholic acid, Simotang, Qingkailing, and senna leaf).

### Data analysis

Normally distributed continuous variables were expressed as the mean ±standard deviation (SD), and groups were compared using the independent t test. Non-normally distributed continuous variables were presented as the median (interquartile range [IQR]), and groups were compared using the rank-sum test. In addition, categorical variables were expressed as numbers (percentages) and analyzed using the chi-square test or Fisher’s exact test. Further, logistic regression models were used to assess independent risk factors for VA-AKI incidence and outcome and death. Multiple logistic regression models were used to identify variables with a P value of less than 0.2 in descriptive analysis; these variables were further examined in multivariate analysis to identify independent risk factors. The covariates included in multiple logistic regression analysis of VA-AKI incidence included sex (male vs female), age (change by 10 years), department (medical, surgical and ICU), CHD (yes vs no), baseline SCr (mg/dL), reason for VAN therapy (prophylactic, local infection and bacteremia), LOS (days), ICU admittance (yes vs no), cancer (yes vs no), concomitant low perfusion factors (yes vs no), receipt of orthopedic/trauma/burn surgery and other concomitant situations during hospitalization (yes vs no), LOT (days), mean daily dosage (change by 0.5 g), and receipt of TDM (yes vs no), vasopressors (yes vs no), nitrates (yes vs no), β-receptor blockers (yes vs no), ACEIs (yes vs no), aminoglycosides (yes vs no), azole antifungals (yes vs no), imipenem-cystatins (yes vs no), diuretics (yes vs no), 20% mannitol (yes vs no), contrast medium (yes vs no), compound glycyrrhizin (yes vs no), ursodeoxycholic acid (yes vs no), etc. A backward logistic model was used for the selection of variables. We used the same method to assess the independent risk factors for death and VA-AKI outcome. All p values were two-sided, and a p value of less than 0.05 was deemed significant. Statistical analyses were conducted using Statistical Package for Social Sciences version 20.0 (IBM, Chicago, Ill., USA).

## Results

### Patients excluded and included

**Fig 2:**
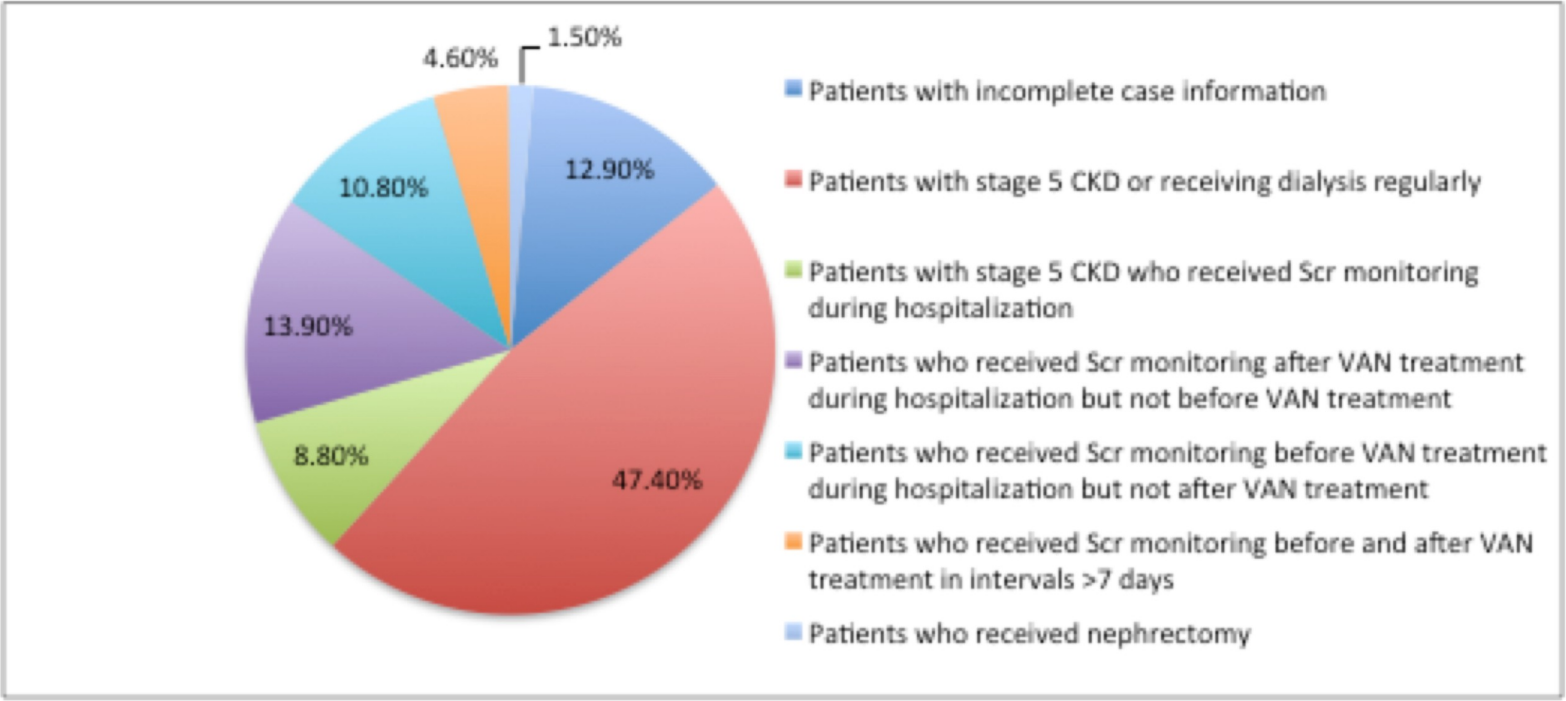
Frequency distribution of excluded patients.

Of the 934 patients who received VAN treatment during their hospital stay, 740 were included in this study. The most common reasons for patient exclusion were the presence of stage 5 CKD or regular receipt of dialysis (47.4%, 92/194) and inadequate SCr monitoring to detect AKI development (38.1%, 74/194)(Fig 2). The total rate of inadequate SCr monitoring among all VAN-treated inpatients was 7.9% (74/934). We further analyzed the departmental distribution of inadequate SCr monitoring, and the results are presented in S1 Table.

A total of 740 patients were included in this study, 50.0% (370/740) of whom were males. The median age was 66 years (IQR = 19). Among the 665 patients included in descriptive analysis, 54.6% (364/665) were females. The ages of 56.2% (374/665) patients were between 60 and 80 years. In addition, 44.4% (291/665) patients concomitant with hypertension, and the rates of concomitant diabetes, CHD, CKD, CLD, cancer and low perfusion factors before or at the time of AKI onset during hospitalization were 22.0% (146/665), 13.7% (91/665), 2.0% (13/665), 2.0% (13/665), and 6.3% (42/665). Further, the median LOS was 25 days (IQR = 14) (Table 1).

**Table 1:** Comparison of clinical characteristics between VA-AKI and No-AKI patients.

	Total n = 665	VA-AKI n = 120	No-AKI n = 545	P value
Demographic factor				
Female, n (%)	364 (54.7)	52 (43.3)	312 (57.2)	0.006
Age (SD), n (%)				0.000
< 50 y	112 (16.8)	29 (24.2)	83 (15.2)	
≥ 50 and < 60 y	91 (13.7)	18 (15.0)	73 (13.4)	
≥ 60 and < 70 y	175 (26.3)	20 (16.7)	155 (28.4)	
≥ 70 and < 80 y	199 (29.9)	26 (21.7)	173 (31.7)	
≥ 80 y	88 (13.2)	27 (22.5)	61 (11.2)	
Weight (SD)*	67.7 ± 12.1	64.2 ± 11.4	68.4 ± 12.1	0.003
Department, n (%)				0.000
Medical	68 (10.2)	22 (18.3)	46 (8.4)	
Surgical	534 (80.4)	67 (56.3)	467 (85.7)	
ICU	62 (9.3)	30 (25.2)	32 (5.9)	
Concomitant diseases				
Hypertension, n (%)				0.659
No hypertension	374 (56.2)	67 (55.8)	307 (56.3)	
Stage 1	72 (10.8)	10 (8.3)	62 (11.4)	
Stage 2	83 (12.5)	18 (15.0)	65 (11.9)	
Stage 3	136 (20.5)	25 (20.8)	111 (20.4)	
Diabetes, n (%)	146 (22.0)	27 (22.5)	119 (21.8)	0.873
CHD, n (%)	91 (13.7)	28 (23.3)	63 (11.6)	0.001
CKD, n (%)	13 (2.0)	4 (3.3)	9 (1.7)	0.401a
CLD, n (%)	13 (2.0)	3 (2.5)	10 (1.8)	0.911a
Laboratory variables				
Baseline SCr, mg/dL	96.0 ± 37.0	120.1 ± 55.3	90.2 ± 28.7	0.000
Reason for VAN therapy, n (%) #1				0.000
Prophylactic	357 (53.7)	14 (11.7)	343 (62.9)	
Local infection	277 (41.7)	87 (72.5)	190 (34.9)	
Bacteremia	31 (4.7)	19 (15.8)	12 (2.2)	
Other risk factors				
Hospitalization-related factors				
LOS (days), n (n)	25 (14)	27 (21)	17 (12)	0.000
ICU admittance, n (%)	109 (16.4)	48 (44)	61 (11.2)	0.000
Cancer, n (%)	42 (6.3)	25 (20.8)	17 (3.1)	0.000
Concomitant ARDS, shock, or low perfusion factors before or at the time of AKI onset during hospitalization, n (%)	42 (6.3)	25 (20.8)	17 (3.1)	0.000
Concomitant hemolysis, rhabdomyolysis or crystallization of urine/urine tube during hospitalization, n (%)	3 (0.5)	1 (0.8)	2 (0.4)	0.450b
Concomitant MOF or DIC during hospitalization, n (%)	12 (1.8)	7 (5.8)	5 (0.9)	0.000
Receipt of endotracheal intubation for ventilator therapy during hospitalization, n (%)	59 (8.9)	40 (33.3)	19 (3.5)	0.000
Orthopedic/trauma/burn surgery during hospitalization, n (%)	379 (57.0)	14 (11.7)	365 (67.0)	0.000
Neurosurgery during hospitalization, n (%)	21 (3.2)	5 (4.2)	16 (2.9)	0.682a
Other surgeries during hospitalization, n (%) #2	160 (24.1)	42 (35.0)	118 (21.7)	0.002

*: n = 592. Only 592 patients’ data on the weight is available. CHD = coronary heart disease, CKD = chronic kidney disease, CLD = chronic liver disease, CEI = angiotensin II-converting enzyme inhibitor, ARB = angiotensin II receptor blocker, NSAID = nonsteroidal anti-inflammatory drug, ARDS = acute respiratory distress syndrome, MOF = multiple organ failure, DIC = disseminated intravascular coagulation.

The VAN used at our hospital was obtained from two sources: Wen kexin and Lai kexin (trade name: Wenkexin, generic name: Vancomycin Hydrochloride for Injection, manufacturer: Eli Lilly KK Seishin Laboratories, specification: 500 mg/bottle; and trade name: Laikexin, generic name: Vancomycin Hydrochloride for Injection, manufacturers: Zhejiang Pharmaceutical Co., Ltd. and Xinchang Pharmaceutical Factory, specification: 500 mg/bottle); 98.6%(655/665) of the patients were treated with VAN from the Wen kexin source. We also surveyed the departmental distribution of the patients receiving VAN treatment, and the results are shown in S2 Table. The reasons for VAN use were mainly prophylaxis and local infection, corresponding to 357 (53.7%, 357/665) and 277 (41.7%, 277/665) patients, respectively. The median LOT was 1 day (IQR = 5), and the median daily dosage was 1.0 g (IQR = 2.0) (Table 2). We also analyzed 38 types of concomitant drugs used by the patients receiving VAN treatment (the details are provided in Table 2).

**Table 2:** Comparison of concomitant drugs used between VA-AKI and No-AKI patients.

	Total n = 665	AKI n = 120	NO-AKI n = 545	P value
Source of VAN, n (%)				1.000b
Wenkexin*1	655 (98.6)	119 (99.2)	536 (98.5)	
Laikexin*2	9 (1.4)	1 (0.8)	8 (1.5)	
LOT, n (n)	1.0 (5.0)	6.0 (11)	1.0 (3.0)	0.000
Mean daily dosage, n (%)				0.000
0.5 g	145 (21.9)	5 (4.2)	140 (25.8)	
1.0 g	273 (41.2)	34 (28.3)	239 (44.1)	
1.5 g	40 (6.0)	13 (10.8)	27 (5.0)	
2.0 g	200 (30.2)	66 (55.0)	134 (24.7)	
3.0 g	4 (0.6)	2 (1.7)	2 (0.4)	
TDM, n (%)	36 (5.4)	19 (15.8)	17 (3.1)	0.000
Concomitant drugs				
Vasopressors, n (%)	56 (8.4)	38 (31.7)	18 (3.3)	0.000
Nitrates, n (%)	69 (10.4)	31 (25.8)	38 (7.0)	0.000
β-receptor blockers, n (%)	156 (23.5)	58 (48.3)	98 (18.0)	0.000
Phentolamine, n (%)	3 (0.5)	3 (2.5)	0 (0.0)	0.006b
Amlodipine, n (%)	123 (18.5)	21 (17.5)	102 (18.7)	0.756
Sodium nitroprusside, n (%)	3 (0.5)	3 (2.5)	0 (0.0)	0.006b
ARBs, n (%)	44 (6.6)	7 (5.8)	37 (6.8)	0.703
ACEIs, n (%)	23 (3.5)	10 (8.3)	13 (2.4)	0.001
Aminoglycosides, n (%)	19 (2.9)	9 (7.5)	10 (1.8)	0.001
Amphotericin B, n (%)	3 (0.5)	1 (0.8)	2 (0.4)	0.450b
Azole antifungals, n (%)	63 (9.5)	30 (25.0)	33 (6.1)	0.000
Micafungin, n (%)	39 (5.9)	20 (16.7)	19 (3.5)	0.000
Metronidazole, n (%)	26 (3.9)	16 (13.3)	10 (1.8)	0.000
Nystatin, n (%)	24 (3.6)	15 (12.5)	9 (1.7)	0.000
Acyclovir, n (%)	24 (3.6)	15 (12.5)	9 (1.7)	0.000
Imipenem-cystatins, n (%)	61 (9.2)	20 (16.7)	41 (7.5)	0.002
β-lactam antibiotics, n (%)	593 (89.2)	103 (85.8)	490 (89.9)	0.193
Macrolides, n (%)	19 (2.9)	11 (9.2)	8 (1.5)	0.000
Quinolones, n (%)	108 (16.2)	35 (29.2)	73 (13.4)	0.000
Sulfonamides, n (%)	175 (26.3)	88 (73.3)	87 (16.0)	0.000
Tigecycline, n (%)	4 (0.6)	2 (1.7)	2 (0.4)	0.310a
Diuretics, n (%)	175 (26.3)	88 (73.3)	87 (16.0)	0.000
20% mannitol, n (%)	24 (3.6)	7 (5.8)	17 (3.1)	0.149
Low-molecular-weight dextran, n (%)	19 (2.9)	0 (0.0)	19 (3.5)	0.033b
NSAIDs, n (%)	222 (33.4)	35 (29.2)	187 (34.3)	0.279
Immunosuppressants, n (%)	20 (3.0)	13 (10.8)	7 (1.3)	0.000
Glucocorticoids, n (%)	122 (18.3)	51 (42.5)	71 (13.0)	0.000
Chemotherapy drugs, n (%)	23 (3.5)	12 (10.0)	11 (2.0)	0.000
Contrast medium, n (%)	22 (3.3)	15 (12.5)	7 (1.3)	0.000
Compound glycyrrhizin, n (%)	98 (14.7)	48 (40.0)	50 (9.2)	0.000
Compound fresh bamboo juice, n (%)	17 (2.6)	6 (5.0)	11 (2.0)	0.061
Liquorice tablets, n (%)	9 (1.4)	1 (0.8)	8 (1.5)	0.914a
Ganmao Qingre granules, n (%)	41 (6.2)	11 (9.2)	30 (5.5)	0.131
Yunnan Baiyao capsules, n (%)	19 (2.9)	11 (9.2)	8 (1.5)	0.000
Ursodeoxycholic acid, n (%)	20 (3.0)	13 (10.8)	7 (1.3)	0.000
Simotang, n (%)	2 (0.3)	1 (0.8)	1 (0.2)	0.329b
Qingkailing, n (%)	2 (0.3)	1 (0.8)	1 (0.2)	0.329b
Senna leaf, n (%)	267 (39.8)	7 (5.8)	258 (47.3)	0.000

*1: Trade name: Wenkexin, generic name: Vancomycin Hydrochloride for Injection, manufacturer: Eli Lilly KK Seishin Laboratories, specification: 500 mg/bottle.

*2: Trade name: Laikexin, generic name: Vancomycin Hydrochloride for Injection, manufacturers: Zhejiang Pharmaceutical Co., Ltd. and Xinchang Pharmaceutical Factory, Specification: 500 mg/bottle.

### TDM of included patients

A total of 5.4% (36/665) of the patients received TDM during VAN therapy. The judgment of whether a patient required TDM was based on《Therapeutic monitoring of vancomycin in adult patients》 [9]. One hundred thirty-seven patients met the requirements for TDM. The total inadequate TDM rate was 73.7% (101/137). We further analyzed the TDM rates for all departments (Table 3). Inadequate TDM was more prevalent in surgical departments, particularly the general surgery department, for which the rate reach the standard was 2.9% (1/35). A total of 35 of 37 patients in the general surgery department required monitoring, only 1 of whom was actually monitored; thus, the actual TDM rate was 2.7% (1/37), which is far less than the recommended rate (94.6%, 35/37). Further, the TDM rate for the patients with a normal baseline SCr was 5.1% (33/641), whereas that for the patients with a baseline SCr of higher than 133 mg/dL was 14.3% (3/21).

**Table 3:** TDM of patients treated with VAN.

Department	Patients who received TDM (n)	Total patients in department (n)	Patients who required TDM (n)	Actual TDM rate	Recommended TDM rate according to guidelines	Rate reach the guidelines
General surgery	1	37	35	2.7%	94.6%	2.9%
Orthopedics	2	387	35	0.5%	9.0%	5.7%
SICU	1	17	14	5.9%	82.4%	7.1%
Neurology	1	6	6	16.7%	100.0%	16.7%
Rheumatism and immunology	1	3	3	33.3%	100.0%	33.3%
Respiratory medicine	8	19	16	42.1%	84.2%	50.0%
Thoracic surgery	1	2	2	50.0%	100.0%	50.0%
CCU	1	2	2	50.0%	100.0%	50.0%
Infectious	1	2	2	50.0%	100.0%	50.0%

CCU = Cardiology Care Unit

Actual TDM rate—patients who received TDM/total no. of patients in department; Supposed TDM rate according to standard—patients who required TDM/total no. of patients in department; Rate reach the standard—actual TDM rate/supposed TDM rate according to standard.

### Clinical characteristics of VA-AKI

A total of 198 patients developed AKI, corresponding to an incidence of 26.8% (198/740). Moreover, 120 patients had confirmed VA-AKI, with an incidence of 16.2% (120/740).

Further, we compared the VA-AKI group with the NO-AKI group (Table 1). The majority of the VA-AKI patients were males (56.7% males vs 43.5% females, p = 0.008); in addition, the median age was 65 years (IQR = 29), and the median weight was 64.2 kg (IQR = 18). The VA-AKI patients were more likely have concomitant CHD (23.3% vs 11.6%, p = 0.000), cancer (20.8% vs 3.1%, p = 0.000), ICU admittance (44% vs 11.2%, p = 0.000) and low perfusion factors before or at the time of AKI onset during hospitalization (20.8% vs 3.1%, p = 0.000). Further, the VA-AKI patients had a longer LOS (27 vs 12 days, p = 0.000) and an elevated baseline estimated glomerular filtration rate (eGFR; 120.1 vs 90.2 mg/dL, p = 0.000). With regard to the reason for VAN therapy, most of the VA-AKI patients were treated for local infection (72.5%, 87/120), whereas the treatment was prophylactic for most of the NO-AKI patients (62.9%, 343/545).

### Concomitant drugs

To further examine the risk factors for the development of VA-AKI, we first further analyzed the concomitant drugs used with VAN. The average daily dosage was 2.0 g for 55.0% (66/120) of the VA-AKI patients, and the median daily dosage was also 2.0 g (IQR = 1). TDM was significantly more commonly performed for the VA-AKI patients (15.8% vs 3.1%, p = 0.000). With regard to the concomitant drugs, compared with the NO-AKI patients, significantly more of the VA-AKI patients received concomitant vasopressors, nitrates, β-receptor blockers, ACEIs, and the following antimicrobial drugs: aminoglycosides, imipenem-cystatins, quinolones, sulfonamides, etc. In addition, the VA-AKI patients were significantly more likely to receive concomitant diuretics, immunosuppressants, glucocorticoids, and contrast medium. We also evaluated the influence of the concomitant use of Chinese patent drugs and found that the VA-AKI patients were more likely to receive concomitant compound glycyrrhizin, Yunnan Baiyao capsules, and ursodeoxycholic acid but were less likely to receive concomitant senna leaf (Table 2).

### Risk factors for VA-AKI

Multiple logistic regression analysis revealed that an elevated baseline eGFR (odds ratio [OR] = 1.009; p = 0.017) and concomitant vasopressor therapy (OR = 2.942; p = 0.009), nitrate treatment (OR = 2.869; p = 0.007), imipenem-cilastatin treatment (OR = 4.708; p = 0.000), and contrast medium administration (OR = 6.609 p = 0.005) were independent risk factors for VA-AKI; in addition, the receipt of orthopedic/trauma/burn surgery (OR = 0.3575; p = 0.011) and concomitant compound glycyrrhizin administration (OR = 0.290; p = 0.017) were determined to be independent protective factors for VA-AKI (See Table 4). **The good of fit was evaluated by the analysis of Hosmer and Lemeshow and the result is 0.652.**

**Table 4:** Risk factors for VA-AKI.

Risk factor	P value	OR	95% CI
Concomitant renal hypoperfusion during hospitalization	0.078	2.193	0.917–5.244
Receipt of endotracheal intubation for ventilation during hospitalization	0.070	2.102	0.942–4.691
Receipt of orthopedic/trauma/burn surgery	0.011	0.357	0.162–0.786
Baseline eGFR	0.017	1.009	1.002–1.016
Vasopressors	0.009	2.942	1.302–6.646
Nitrates	0.007	2.869	1.331–6.185
Imipenem-cilastatin	0.000	4.708	2.611–8.489
Contrast medium	0.005	6.609	1.732–21.267
Compound glycyrrhizin	0.017	0.290	0.105–0.800
Ganmao Qingre granules	0.061	2.670	0.955–6.463

### VA-AKI severity and treatment

VA-AKI severity was classified according to the highest stage of AKI observed in the patient and receipt of RRT. The highest disease stage was stage 1 for 69.2% (83/120) of the patients, and 2.5% (3/120) of them received RRT (S3 Table). The median time of peak disease severity was 5 days (IQR = 6) after initiation of VAN therapy. Next, we analyzed the departmental distribution of the VA-AKI patients. We excluded the patients with VA-AKI caused by preventive administration of VAN from this analysis because this group of patients consistently exhibited a better baseline condition and lower LOT of VAN treatment. For the purpose of therapy, the departments with highest VA-AKI incidences were the cardiac surgery department, orthopedics department and bone marrow transplant ward, with incidence rates of 59.3% (16/27), 50% (20/40) and 48.2% (13/27), respectively. Therapy adjustments, including termination of VAN treatment or a decrease in the dosage, were only made for 40.8% (49/120) of these VA-AKI patients after the onset of AKI (Table 5).

**Table 5:** VA-AKI treatments characteristics.

Treatment	n (%)
No adjustment	68 (56.7)
Dose adjustment or other protective measures*	29 (24.1)
Stop/replace VAN	20 (16.7)
Renal replacement therapy	3 (2.5)

*protective measures include: addition of renal protective drugs, TDM for VAN, RRT, etc.

### Outcomes and risk factors for VA-AKI

The all-cause in-hospital-mortality rate for the VA-AKI patients was 18.33% (22/120), and 76.2% (17/22) had an eGFR that was outside of the normal range at the time of death, suggestive of some degree of kidney injury (Table 6). Multiple logistic regression analysis revealed that the independent risk factors for death for the total VA-AKI patients were CHD (OR = 12.6; p = 0.006) and concomitant vasopressor therapy (OR = 15.4; p = 0.001) (Table 7). We separated the renal outcomes of the VA-AKI patients into three categories: recovery, improvement and non-recovery. Renal function recovered in 11 patients, with a median recovery time of 4 days (IQR = 6) after receipt of VAN therapy and renal function improved in 17 patients. Thus, 28 patients showed either recovery or improvement, representing 23.3% (28/120) of the total VA-AKI patients. The remaining 73.3% (92/120) of the VA-AKI patients had renal insufficiency at the time of hospital discharge. Multiple logistic regression analysis revealed that concomitant CHD (OR = 8.858, p = 0.019) and contrast medium administration (OR = 9.779, p = 0.005) were independent risk factors for VA-AKI and that β-receptor blocker treatment (OR = 0.124, p = 0.001) was an independent protective factor (see Table 8). We further performed an additional post-estimation analysis with Akaike score and a table of the classification of the model (Table 9).

**Table 6:** VA-AKI patients’ renal function at the time of death.

Renal function (eGFR) ml/(min·1.73 m^2^)	n (%)
1. ≥90	5 (23.8)
2. ≥60且<90	0 (0)
3. ≥30且<60	5 (23.8)
4. ≥15且<30	8 (38.1)
5. <15 or dialysis	3 (14.3)

eGFR = 175 × [SCr (mg/dl)]-1.234 ×[age(y)]-0.179 ×sex(male = 1, female = 0.79).

**Table 7:** Risk factors for death in VA-AKI patients.

Factors	P Value	OR	95% CI
CHD	0.006	12.6	2.1–77.1
Vasopressor use	0.001	15.4	3.2–75.7
Contrast medium administration	0.998	0.000	0.000

**Table 8:** Risk factors for renal outcome in VA-AKI patients.

Factor	P Value	OR	95% CI
CHD	0.019	8.858	1.442–54.406
LOS	0.076	1.015	0.998–1.032
Reception of other surgeries during hospitalization	0.082	3.171	0.865–11.623
β-receptor blocker	0.001	0.124	0.062–0.881
20% mannitol	0.998	0.000	0.000
Contrast medium	0.005	9.779	1.957–48.864
Compound glycyrrhizin	0.083	0.307	0.081–1.164
Yunnan Baiyao capsules	0.998	0.000	0.000

**Table 9:** Akaike score and a table of the classification of the model.

Goodness of fit evaluation	Risk Factors For VA-AKI	For Death	For Renal Outcome
Akaike score	405.233	95.222	112.233

## Discussion

VAN is a key antibiotic used for the management of severe Gram-positive infections, particularly MRSA infections. However, VAN treatment failures have occurred in patients infected with MRSA isolates reported to be associated with the development of severe infections. The use of a higher VAN concentration has been recommended, which necessitates evaluations of drug safety, especially nephrotoxic and ototoxic agents. The optimal use of VAN is still a complicated issue [1–3]. This single-center retrospective study conducted at our hospital aimed to investigate the current situation of VA-AKI in China and identify risk factors for VA-AKI to provide some suggestions for improving the prevention and treatment of AKI.

To confirm the occurrence of VA-AKI in this population, we designed a four-step survey abiding by the principles of broad screening and enrollment but utilizing strict identification criteria (Fig 1). During the process of confirming VA-AKI diagnoses, we found that a subset of the VAN-treated patients received insufficient SCr monitoring (7.9%).,Greater attention must be paid to the monitoring of SCr in patients receiving VAN, especially the surgery department, to reduce the misdiagnosis of AKI. The rate of AKI detection was 26.6% and the incidence of VA-AKI was 16.2% at our hospital. Nobutoshi Masuda has reported that intervention by a pharmacist might impact VAN therapy, as it might enable the balancing of a higher trough VAN concentration with the risk of nephrotoxicity. In that study, pharmacists monitored renal function by measuring patients’ SCr levels, and intervention by a pharmacist was determined to be associated with a 45% decrease in the incidence of nephrotoxicity[10]. Kathleen A. Marquis’ study reached similar conclusions, showing that a pharmacist-directed VAN pilot program significantly increased the percentage of patients who were optimally dosed. Patients in the pilot program had a shorter LOT and lower incidence of nephrotoxicity [11]. We recommend that hospitals increase their investment in clinical pharmacists because pharmacists not only enable clinicians, especially surgeons, to focus on their work but also more professionally manage VAN treatment.

In further analyses comparing NO-AKI patients with VA-AKI patients, we found out that the VA-AKI patients were significantly more likely to have CHD as an underlying disease; however, no significant differences in the incidences of other underlying diseases were observed between these groups. Further, the VA-AKI patients were significantly more likely to have an elevated baseline eGFR, longer LOS, higher rate of ICU care and higher rate of concomitant disease. Moreover, the VA-AKI patients had more serious conditions than the NO-AKI patients, and more severe comorbidity is often correlated with a higher risk of nephrotoxicity. This study also revealed that an elevated baseline eGFR was an independent risk factor for the development of VA-AKI, consistent with the findings of a previous study by Brady S. Moffett but in contrast with the results of studies by Lindsey Pritchard and Stephen W. Davies [12–14]. Our explanation for these findings is that clinicians may pay more attention to patients with insufficient renal function. For example, the TDM rate for the patients with a normal baseline SCr was (5.1%) lower than that for the patients with a baseline SCr of higher than 133 mg/dL (14.3%).

We more extensively and more elaborately studied concomitant drugs used during or before VAN treatment compared with previous studies. In addition, this was the first such study to include the concomitant use of Chinese patent drugs. The VA-AKI patients in this study were significantly more likely to receive concomitant compound glycyrrhizin, Yunnan Baiyao capsules, and ursodeoxycholic acid and were less likely to receive concomitant senna leaf. Studies conducted in China have demonstrated that compound glycyrrhizin, Yunnan Baiyao capsules, and senna leaf provide a certain degree of improvement in renal function, but their specific underlying mechanisms require further investigation [15–17]. The sub-classification of drugs in this study was also more detailed compared with previous studies. Many studies had shown that the use of vasoactive drugs is an independent risk factor for the development of VA-AKI [12,18]. The term vasoactive drug covers a wide range of drugs, and in our study, we subdivided vasoactive drugs into 8 specific classes (vasopressors, nitrates, β-blockers, phentolamine, calcium channel blockers, sodium nitroprusside, ARBs, and ACEIs). Multivariate logistic regression analysis revealed that concomitant vasopressor use was an independent risk factor for VA-AKI, consistent with Kathleen A. Hazlewood’s study [4]. The use of vasopressors often indicates that patients have severe concomitant disease symptoms. This is the first study to confirm that concomitant nitrate use is an independent risk factor for VA-AKI. The reason for this finding might be that previous studies did not subcategorize vasoactive drugs into this specific class. Nitrate drugs themselves do not have kidney toxicity. We found that when a patient was using nitrates, he (she) was more likely to concomitant with CHD (49.3%, 34/69 vs 9.6%, 57/596) and hypertension. CHD was reported to be a close connection with worsening renal function [19,20]. Multiple logistic regression analysis also showed that the concomitant use of imipenem-cystatins was an independent risk factor for VA-AKI, in disagreement with most current studies. Blanca Humanes’ study has demonstrated that cilastatin protects against VAN-induced proximal tubule apoptosis and increases cell viability without compromising the antimicrobial effects of VAN. The beneficial effects of cilastatin could be at least partly attributed to the decreased accumulation of VAN in renal proximal tubular epithelial cells [21]. In our study, we assessed imipenem-cystatins rather than cystatins, as there is no “cystatin” drug available in China. Notably, our results may have been influenced by the fact that patients taking imipenem-cystatins always have a concomitant severe infection. In conclusion, greater attention must be paid to patients receiving VAN together with vasopressors, nitrates and/or contrast medium.

Multiple logistic regression analysis revealed that the concomitant use of compound glycyrrhizin was an independent predictor of VA-AKI; this study is the first to report this finding. Compound glycyrrhizin is a preparation composed of β-glycyrrhizic acid glycosides, glycine, methionine and other compounds [22]. Experiments using rats have confirmed that glycyrrhizic acid inhibits the development of renal interstitial fibrosis [23]. We have also observed that compound glycyrrhizin significantly reduces proteinuria and enhances renal function in CKD patients [16]. Receipt of orthopedic/trauma/burn surgery is a prospective factor in our study, but its clinical significance may not be very important. The reason to this statistics result may be that the majority of the patients who received orthopedic/trauma/burn surgery have a better baseline condition, a younger age, shorter LOS, lower rate of ICU stay, less concomitant drugs and so on. All this factors contributed to the result that this group of patients would be less likely to develop AKI and contributed this statistics result further.

### Risk factors for mortality and other outcomes

A total of 120 patients developed VA-AKI, and their mortality rate was 18.3% (22/120). The mortality rate for all patients included in this study was 6.1% (45/740). Thus, the mortality rate for AKI patients was higher than that for NO-AKI patients. In addition, the LOS of the AKI patients were significantly longer than that of the NO-AKI patients (27 vs 17, p = 0.000). Chertow GM’s study has also shown that AKI development is associated with increases in the mortality rate and LOS for these patients [24]. In this study, univariate logistic regression analysis revealed that AKI development was a risk factor for mortality (p = 0.000). Because only a small number of patients died (n = 45), it was difficult to further determine whether the occurrence of VA-AKI was an independent risk factor for death in our study. Thus, we could not establish a causal link between AKI and death. Similarly, P. Hanrahan’s study was not able to demonstrate that AKI was an established independent risk factor for increased mortality [25]. However, Glaucia T. F. Seixas’ study and some other published studies have consistently shown that nephrotoxicity is an independent risk factor for death, even after adjusting for comorbidities and disease severity [26]. Furthermore, our multiple logistic regression analysis demonstrated that concomitant CHD (OR = 12.6; p = 0.006) and vasopressor therapy (OR = 15.4; p = 0.001) were independent risk factors for death. Thus, greater attention must be paid to patients using VAN who have concomitant CHD or are simultaneously receiving vasopressor therapy.

Among the patients treated with VAN who developed AKI, 56.7% (68/120) did not receive any targeted treatment, and 2.5% (3/120) received RRT. The type of treatment may be related to AKI severity. The highest AKI stage was stage 1 in 70.8% (85/120) of the patients and stage 3 in 15.0% (18/120) of the patients. For mild cases of AKI, our first consideration is drug dosage adjustment and other renoprotective measures; RRT is used only in severe cases.

We further analyzed the outcomes of AKI. Renal function improved in 23.3% (28/120) of the AKI patients, but 73.3% (92/120) of the patients left the hospital with renal insufficiency. This is a very promising finding. Following the development of AKI, the failure to correct renal function in time would not only increase patient suffering but also increase the LOS and cost of treatment. More importantly, long-term renal insufficiency might promote the progression of AKI to CKD. The consequences of such an outcome are difficult for us to convey. Multiple logistic regression analysis revealed that CHD (OR = 8.858, p = 0.019) and contrast medium administration (OR = 9.779, p = 0.005) were independent risk factors for VA-AKI. The improvement of renal function is more difficult in patients with concomitant CHD and contrast medium administration. Thus, greater attention must be paid to AKI patients with concomitant CHD, and contrast medium should be more carefully administered to these patients. Multiple logistic regression analysis also showed that β-receptor blocker use (OR = 0.124, p = 0.001) was an independent protective factor for AKI. However, the specific mechanism underlying this effect is unclear and warrants further investigation.

### Strengths and limitations

The strengths of this study are as follows. First, this study examined a large Chinese population, and previous studies of risk factors for AKI in the Chinese population are very limited. Second, our survey of AKI was designed to include four steps, enabling not only analysis of the situation of AKI and its associated risk factors but also the treatment and outcomes of this condition. In this study, we identified risk factors for VA-AKI to support the prevention and management of AKI. Third, our study analyzed different types of drugs used concomitantly with VAN treatment; the diversity of drugs examined in this study is greater than that in most previous studies, and this is the first study to examine the concomitant use of Chinese patent drugs. However, this descriptive study has several limitations. First, this was a single-center retrospective study. Second, we lacked VAN therapeutic concentrations due to the low rate of TDM in clinical practice in the real word. Third, our study did not analyze the plasma VAN concentration, SCr concentration, or LOS as risk factors for AKI. Fourth, our study did not analyze the cost of AKI.

## Conclusion

A subset of patients treated with VAN received insufficient SCr monitoring and clearly inadequate TDM. We recommend that hospitals increase their investment in clinical pharmacists. An elevated baseline eGFR and concomitant vasopressor therapy, nitrate use, imipenem-cilastatin use, and contrast medium administration were independent risk factors for VA-AKI; in addition, the receipt of orthopedic/trauma/burn surgery and concomitant use of compound glycyrrhizin were independent protective factors for VA-AKI.

## Supporting information

S1 Table Departmental distribution of inadequate SCr monitoring (DOCX)

S2 Table Departmental distribution of patients receiving VAN therapy (DOCX)

S3 Table The severity of VA-AKI. (DOCX)

S1 File PUFH clinical research ethics committee approval file (PDF)
