# Supplementary material for: Vancomycin-associated acute kidney injury: A cross-sectional study from a single center in China
Source: PLoS One. 2017 Apr 20;12(4):e0175688. doi: 10.1371/journal.pone.0175688 (PMC5398886; doi:10.1371/journal.pone.0175688)
Supplement: S1 Table — (DOCX) [file pone.0175688.s001.docx]

**Supplemental Table 1. Departmental distribution of** **inadequate SCr monitoring**

| Department | n | % |
| --- | --- | --- |
| Urology | 21 | 28.4% |
| Neurosurgery | 15 | 20.3% |
| Orthopedics | 6 | 8.1% |
| Infection | 5 | 6.8% |
| General surgery | 5 | 6.8% |
| Respiratory medicine | 4 | 5.4% |
| Thoracic surgery | 3 | 4.1% |
| Cadre ward | 2 | 2.7% |
| Respiratory medicine ICU | 2 | 2.7% |
| Chemotherapy | 2 | 2.7% |
| Nephrology | 2 | 2.7% |
| Surgical ICU | 2 | 2.7% |
| Ophthalmology | 2 | 2.7% |
| Bone marrow transplantation ward | 1 | 1.4% |
| Burn surgery | 1 | 1.4% |
| Cardiac surgery | 1 | 1.4% |
