# Supplementary material for: Vancomycin-associated acute kidney injury: A cross-sectional study from a single center in China
Source: PLoS One. 2017 Apr 20;12(4):e0175688. doi: 10.1371/journal.pone.0175688 (PMC5398886; doi:10.1371/journal.pone.0175688)
Supplement: S2 Table — (DOCX) [file pone.0175688.s002.docx]

**Supplemental Table 2.** **Departmental Distribution of Patients Receiving VAN Therapy**

| Department | N | % |
| --- | --- | --- |
| Orthopedics | 387 | 58.2% |
| General surgery | 37 | 5.6% |
| Neurosurgery | 31 | 4.7% |
| Cardiovascular surgery | 29 | 4.4% |
| Bone marrow transplantation | 27 | 4.1% |
| Cardiac surgery | 20 | 3.0% |
| Respiratory medicine ICU | 18 | 2.7% |
| Respiratory medicine | 18 | 2.7% |
| Surgical ICU | 17 | 2.6% |
| Urology | 10 | 1.5% |
