# Supplementary material for: Vancomycin-associated acute kidney injury: A cross-sectional study from a single center in China
Source: PLoS One. 2017 Apr 20;12(4):e0175688. doi: 10.1371/journal.pone.0175688 (PMC5398886; doi:10.1371/journal.pone.0175688)
Supplement: S3 Table — (DOCX) [file pone.0175688.s003.docx]

**Supplemental Table 3.** **The severity of VA-AKI**

| Severity | N | % (N/120) |
| --- | --- | --- |
| Stage 1 | 83 | 69.2% |
| Stage 2 | 17 | 14.2% |
| Stage 3 | 17 | 14.2% |
| RRT | 3 | 2.5% |
