# Supplementary material for: Vancomycin-associated acute kidney injury: A cross-sectional study from a single center in China
Source: PLoS One. 2017 Apr 20;12(4):e0175688. doi: 10.1371/journal.pone.0175688 (PMC5398886; doi:10.1371/journal.pone.0175688)
Supplement: S1 File — (PDF) [file pone.0175688.s004.PDF]

北京大学第一医院临床研究伦理委员会

批　准　书

批准号：2015[998]

项目名称：万古霉素导致急性肾损伤与基因相关性研究	
项目负责人：崔一民	职称：教授
研究单位：北京大学第一医院	研究科室：药剂科
合作单位：北京大学第一医院肾内科	
研究时间：2015 年 12 月 至 2016 年 12 月	
项目来源：科室自筹	

评审意见：

研究递交的审查材料：

研究方案版本号及日期：　Version.2　2015/12/01

知情同意书版本号及日期：Version.2　2015/12/01

经伦理委员会审查，符合伦理学要求。

北京大学第一医院临床研究伦理委员会

（印章：北京大学第一医院 临床研究伦理委员会）

主任委员：张岩

2015 年 12 月 15 日
